# La Grippe or Russian influenza: Mortality statistics during the 1890 Epidemic in Indiana

**DOI:** 10.1111/irv.12632

**Published:** 2019-02-12

**Authors:** E. Thomas Ewing

**Affiliations:** ^1^ Department of History Virginia Tech Blacksburg Virginia

**Keywords:** epidemic, epidemiology, history, influenza, mortality

## Abstract

**Background:**

The Russian influenza, which began in late 1889, has long been recognized as a major global epidemic yet available statistical evidence for morbidity and mortality has not been fully examined using historical and epidemiological tools. This study of cases and deaths in Indiana during the extended time period associated with the Russian influenza is the first scholarly effort to determine the number of victims from this influenza outbreak across a broad regional case study in the US.

**Methods:**

The sources for this study include historical records from the US Census, Annual Reports from the Indiana State Board of Health, and death notices published in newspapers. The available evidence is analyzed using historical and epidemiological methods to determine the consistency of reporting categories, the accuracy of death records, and the applicability of contemporary categories for measuring mortality.

**Results:**

In the 3 years during and following the outbreak of “Russian influenza” in January 1890 in the state of Indiana, approximately 3200 died specifically of this disease while a total of 11 700 died of influenza and other respiratory diseases. These results confirm that extremely widespread influenza contributed to higher than normal death rates by causing additional deaths in related categories, especially pneumonia and other respiratory diseases.

**Conclusions:**

More reliable and thorough analysis of morbidity and mortality during the Russian influenza based on systematic and critical review of local, regional, and national statistics can inform contemporary understanding of the long‐term history of influenza epidemics.

## 1 INTRODUCTION

On January 26, 1890, the *Indianapolis Journal* newspaper noted the “steady increase in the city death rate for the past 4 weeks.”^1^ In fact, the weekly total of deaths had doubled, from 28 at the start of the month to 55 in the preceding week. The “sole cause” for this “sudden increase” was reported to be influenza “and its various complications.” Of the 169 total deaths reported in January, more than 20% “have been due to complications of influenza.” In addition to these quantitative measures, the article provided a list of victims, most with ages and addresses, classified as “deaths here from simple and aggravated cases of influenza.”

One listed victim was John Bussey, a saloonkeeper, who died at age 62 on January 2, 1890. Yet this man's death, and the way it was reported in the newspaper, illustrates how counting influenza deaths was problematic even at the level of individual victims. On January 3, the *Indianapolis Journal* reported that Bussey died at his home “from what was supposed to be the influenza,” after “suffering from the disorder for a few days.”^2^ But this same article used Bussey's death to highlight the debate among “local physicians as to whether or not la grippe can actually claim any victims in the city, it believed by some that no case had yet appeared in the State.” The latter position was affirmed by Marion County Coroner Dr. T. A. Wagner: “I would be willing to put up fifty dollars that there is not a single case in the city. Those physicians who claim they have patients suffering from it are only mistaken in their diagnoses.”

**Figure 1:**
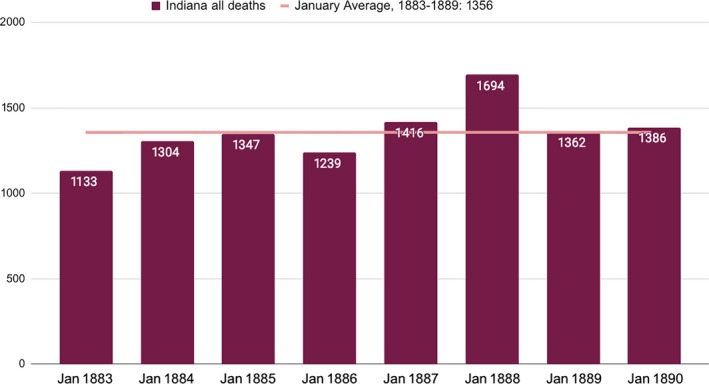
Indiana, Deaths from all causes, January 1883‐1889^3^, ^4^, ^5^, ^6^, ^7^, ^8^, ^9^, ^10^

Although these newspaper articles illustrated growing awareness about an unusual number of influenza cases and deaths, the Indiana Board of Health Annual Report indicated that the total number of deaths in January 1890 was actually quite consistent relative to previous years (Figure 1).^3^, ^4^, ^5^, ^6^, ^7^, ^8^, ^9^, ^10^ The total of 1386 deaths in January 1890 was only 2% higher than the January average for the years 1883‐1889. As will be discussed more fully below, these Board of Health numbers, although detailed, represented only one‐half of total deaths in the state.

These three examples—the death of John Bussey, a newspaper article calling attention to increased death totals, and statistical data comparing successive years—illustrate both the challenges and the importance of determining the actual impact of any disease outbreak, but particularly an influenza epidemic. This analysis focuses on the tensions embedded in these three reports, as the confidence (and denial) expressed by physicians and health officials related to Bussey's death can be compared to the more alarmist tone of the newspaper article calling attention to the sudden change in the number of deaths, even as the long‐term perspective from annual reports suggests that these fears may have been exaggerated.

Understanding the number of cases and deaths during the epidemic that began in 1890 matters because this outbreak is one of the major influenza epidemics of the last two centuries, occurring at a moment when physicians and officials recognized vital statistics as a tool to improve public health, yet were still working out methods for measuring morbidity and mortality. This approach takes a unique approach to historical epidemiology by examining all available data about cases and deaths within a defined geographical space. The study of historical epidemics faces many of the same challenges as contemporary efforts to document influenza‐associated deaths, including the importance of distinguishing excess mortality during severe outbreaks from typical mortality caused by seasonal influenza, the lack of reliable tests confirming influenza as cause of death for individuals, the potential for underlying structural causes to affect mortality levels, and the importance of counting deaths for which influenza may be an associated, but not direct, cause of death. These interpretive challenges are evident in the varied terminology used in current studies, including influenza‐associated mortality, deaths from respiratory and circulatory diseases or pneumonia and influenza, and cases of influenza‐like illness.^11^, ^12^, ^13^, ^14^, ^15^, ^16^ Given these challenges, which are even more complicated for a historical study, this article integrates a wide range of publicly available data on diseases and deaths, including the 1890 US Census (available from the US Census Bureau), the Indiana State Board of Health annual reports (available from Hathi Trust library), and articles about death rates and individual victims in newspapers (available in digital newspaper collections).

The objective of this study was to contribute to scholarship on morbidity and mortality during the 1890 influenza epidemic, thus building upon scholarship that documents death rates during historical epidemics. While most research on the Russian influenza has focused on the spread of disease, the response of medical authorities, and the cultural experience of an epidemic,^17^, ^18^, ^19^, ^20^, ^21^, ^22^ the few efforts to measure the impact of the epidemic have relied on a limited number of statistical reports drawn mostly from major cities, which are then generalized to make broader claims.^23^, ^24^, ^25^, ^26^ This study, by contrast, explores all of the available evidence for a particular region, while also recognizing the need to think critically about the origins, nature, and implications of the statistical information collected during and after the epidemic to enhance understanding of both this historical example and broader challenges of influenza epidemiology. This approach illustrates important similarities with the Spanish influenza almost three decades later, including the rapid spread of the disease and the unexpected impact on death rates, while also acknowledging the much higher mortality rates that began in the fall of 1918.^24^, ^25^, ^26^, ^27^, ^28^, ^29^, ^30^

**Figure 2:**
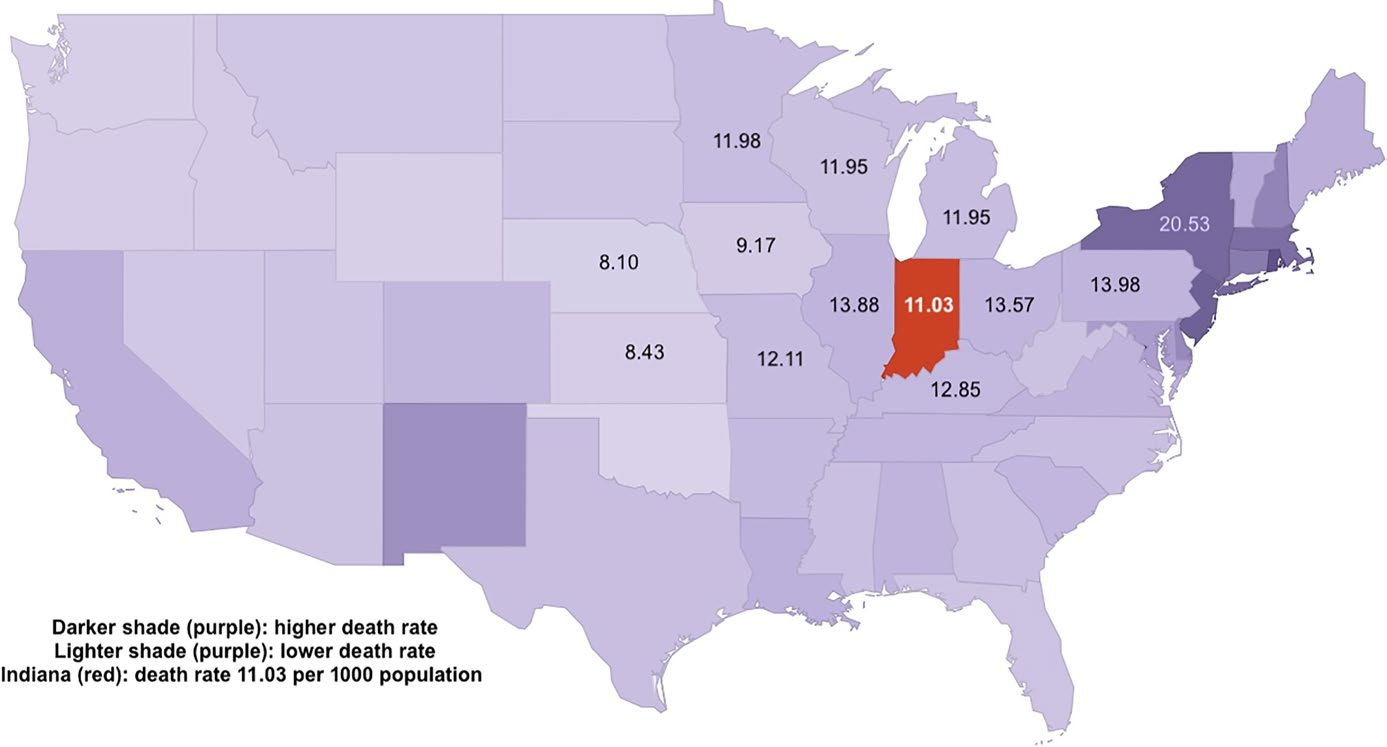
Death Rate, all causes, compared to Indiana, 1890 Census^32^

Indiana is selected for this case study because the population was broadly representative of the US but also because the available data make it possible to engage in thoughtful analysis of influenza cases and deaths. According to the 1890 census, Indiana was the eighth most populous state, with a population of just under 2.2 million, which accounted for 3.5% of the US population of 62 million.^31^ Indiana ranked tenth in the US in terms of total deaths, according to the 1890 census, but thirty‐third in terms of death rates, with a much lower rate (11.03 deaths per 1000 population) than states with larger populations on the eastern coast, but broadly similar to the states also located in this region of the country (Figure 2).^32^ Given these patterns, Indiana can serve as a representative case study with a substantial population and a death rate consistent with comparably sized and located states.

Influenza only appears as a cause of death in one table in the 1890 census with statistics from all states: Table 18, which includes “La Grippe” as one of 137 specific causes listed alphabetically but not grouped by any classifications, for the census year, which began on June 1, 1889 and ended on May 31, 1890.^32^ According to this table, 630 deaths were attributed to influenza in Indiana, compared to 12 957 deaths in the US. In Indiana, influenza accounted for 2.6% of all deaths with a death rate of 0.29 deaths per 1000 population. In the US, influenza accounted for 1.5% of all deaths with a death rate of 0.21 deaths per 1000 population. In terms of both percentage of all deaths and death rate, influenza accounted for proportionally more deaths in Indiana than in the US as a whole.

Indiana Board of Health Reports provide a different number for total deaths during the epidemic.^10^ The state reported deaths by fiscal year, so the 1890 Annual Report attributed 388 deaths to “La Grippe” from October 1, 1889, to September 30, 1890, which included the peak weeks of the epidemic in January‐March 1890. In addition, the 1890 Annual Report attributed two deaths to “Influenza,” listed separately from “La Grippe.” For the census year, from June 1, 1889, to May 31, 1890, the combined statistics from the 1889 and 1890 annual reports indicate 343 deaths from influenza.^10^, ^33^ Finally, during the calendar year 1890, the combined statistics from the 1890 and 1891 reports indicated 403 deaths from influenza. All four of these numbers—343, 388, 403, and 630—are documented answers to the question: How many people died in Indiana from influenza during the 1890 epidemic?

The most obvious explanation for the difference between the higher census number and the lower state totals is the fact that the Board of Health statistics were admittedly incomplete. According to the 1890 Annual Report, “not one‐half of deaths are reported” by county health officers.^10^ In fact, a comparison for the census year indicates that the Board of Health statistics accounted for just 62% of deaths reported in the census. This ratio was consistent across respiratory diseases, including la grippe (54%), phthisis (55%), pneumonitis (58%), and bronchitis (60%). In this sense, the US census total is actually confirmed by the State Board of Health, even as it reported just one‐half the number of deaths.

Yet the challenges of counting influenza deaths began at the level of individual victims, as suggested by the example of Bussey cited above. Newspaper obituaries and death notices occasionally identified influenza or la grippe as the primary cause of death: Jacob Kiefer “died from influenza after an illness of two days,” Minnie Arnold died of “la grippe,” and John Wood died of “old age and influenza” after being sick “for some time.”^34^, ^35^, ^36^, ^37^ In other cases, influenza or la grippe was paired with another disease. Robert Bence died from “a complication of influenza and pneumonia,” Carrie Garnett, an African American woman, died of “chronic bronchitis and influenza,” Charles Howard, less than 2 years old, died of “inflammation of the brain and la grippe,” and Jesse Burdett, “died from heart disease a la grippe.”^38^, ^39^, ^40^ The deaths of these individuals, as reported by newspapers, illustrate both the human costs of this disease and the challenge of identifying cause of death in ways that provide a meaningful and consistent basis for quantified analysis.

**Figure 3:**
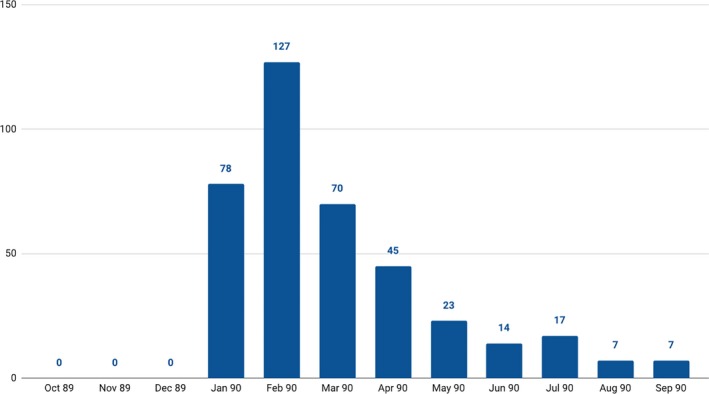
La Grippe Deaths in Indiana, 1890 annual report^10^

Whereas the cause of death illustrated ambiguity in reporting categories, the sudden spike in deaths attributed to influenza (Figure 3)^10^ was readily apparent at the time to observers and subsequently confirmed by the State Board of Health. According to monthly reports for the state, the spike in influenza deaths in the first 3 months of 1890 accounted for more than two‐thirds of deaths from this cause during the entire year. Unfortunately, because the State Board of Health only began to record La Grippe deaths following the outbreak in January 1890, it is not possible to make direct comparisons to this cause of death in previous years.

The perception of the influenza epidemic as a substantially different moment in public health was illustrated in the opening section of the Board of Health Annual Report for 1890, published toward the end of the year: “During the latter part of last year and the first of this year La Grippe or Russian influenza made its appearance and swept over the entire State.”^10^ The report declared that while “the mortality directly traceable to the disease was comparatively light,” many victims were “in such a debilitated condition that many deaths occurred from sequelae of the attack.” The report conceded, however, that efforts “to get complete statistics concerning the disease” had not been successful, in part because few physicians actually maintained records of cases or deaths, leading to the following conclusion: “Only about 400 deaths are reported from this cause, which number is doubtless too small, but the exact number cannot be known.”^10^

Reports from county health officers provide further evidence for the importance, but also difficulties, of measuring the impact of the epidemic.^10^ Of the 36 reports published by the State Board of Health for 1890 (more than one‐third of all counties), 30 specifically referenced the La Grippe epidemic. Many county health reports acknowledged the unusual extent and even severity of the epidemic, often expressed in vivid imagery. Dr. V. H. Gregg, Health Board Secretary in Fayette County, reported that “influenza (la grippe) in the winter of 1889 and 1890” had epidemic characteristics: “reaching from ocean to ocean, and spreading from continent to continent, like a chilly wave, casting a gloom of awe over us for a few days, leaving scarcely a trace of its ravages, except a sequela of some few chronic cases of tuberculosis or rheumatic diathesis.” Dr. W. V. Wiles, Health Officer for Owen County, offered a colorful description of “the ravages of that new enemy of the human family, la grippe” which first appeared in November 1889:

During the first three months, only a small proportion of the population was affected, but within the next three months, fully four‐fifths of the people, of all classes, had become affected. It approached its victims, as a rule, without warning or premonitory symptoms and prostrated them as unceremoniously as an expert sandbagger would fell the belated and weary pedestrian.

This sense of a widespread epidemic was consistent with emphatic, although imprecise, statements about the proportion or number of victims: 80% of the population in Owen and Warrick Counties, a “majority of the people” in Henry County, a “large majority” in Tippecanoe County, “all over” Boone and Newton Counties, “almost universal” in Kosciusko and Putnam Counties, “nearly everybody was sick with it” in Fulton County, and “quite a number” in Jay County. By contrast, many fewer reports described the epidemic as having only a limited effect. In Lake County, “very few cases of ‘La Grippe’ [were] reported; most of them recovered.” In Ripley County, the “epidemic of la grippe, although involving many in its wicked grasp, disappeared without doing much damage to the public health.”

Just four county health reports included specific information about the number of victims: “almost three hundred cases of la grippe” in Adams County, 3461 cases in Hendricks County, 3000 cases in Clinton County, and 7396 cases in Wayne County. Census data^31^ make it possible to calculate a very wide range of morbidity rates per 1000 population: 14.87 in Adams County, 109.61 in Clinton County, 160.99 in Hendricks County, and 196.56 in Wayne County.

Reporting on deaths offered a similar combination of limited numerical data together with broad statements, with the latter consistently claiming low mortality rates. In Montgomery County, “notwithstanding the number affected by this disease, the mortality was small.” In Porter County, influenza produced “very few, if any, deaths.” In Whitley County, “the disease was very mild in the great majority of cases; indeed, a great many cases were observed which did not require any treatment, and only a few deaths were attributed to this disease.”

County health reports confirmed the evidence from individual victims cited above regarding the higher death rate when complicated by other health conditions. Dr. J. F. Beckner stated that la grippe was “general all over” in Newton County: “But few deaths occurred at the time of its prevalence, but it caused many deaths throughout the county by awakening latent diseases, such as tuberculosis and rheumatism, and a few cases of insanity that were caused by la grippe.” Putnam County Health Board Secretary G. W. Bence offered a detailed account of the epidemic's impact:

The immediate death rate was small for so severe and general an epidemic, but the remote effects are still felt. Many cases of incipient phthisis were precipitated; hence the deaths from phthisis are increased in number, and in many persons the system was left so debilitated that attacks of the most common diseases assumed a more severe character and were more difficult to control.

Vigo County Health Officer Leo J. Weinstein attributed no deaths to influenza, “so far as I know,” but he added this qualification: “Of course many died from other causes during the epidemic that would otherwise not have done so.”

The few county reports that provided statistics on the number of deaths offer important insights into the impact of the epidemic. In Hendricks County, 3461 cases resulted in just 26 deaths, and “most of these were suffering from chronic disease.” Clinton County recorded four deaths from influenza and four more deaths from “diseases complicated with la grippe.” In Lagrange County, influenza caused only two or three deaths, out of more than seventy deaths from all causes. In White County “directly or indirectly perhaps a dozen deaths were caused by the above‐named disease, and its effects are still felt by many.” In Randolph County, seven deaths were attributed to influenza and three deaths to la grippe, accounting for 8% of all deaths.

Do county reports provide a basis for estimating the number of influenza victims in Indiana? The answer, and thus the reliability, depends on which numbers are used for making estimates. Using the reported rate for Wayne County, one in four, would suggest that more than 500 000 people in Indiana suffered from influenza in early 1890. Using reports from other counties would produce completely different estimates of the number of cases: The morbidity rate of 14.87 reported from Adams County would indicate just 32 000 victims, whereas the rate of 160.99 in Hendricks County would indicate 354 000 victims. If 80% of the population was ill, a rate suggested by statements that “nearly everybody” was sick and the disease was “almost universal,” the total victims could have been 1.75 million.

Given this range of potential morbidity, estimates of case fatality rate (CFR) will be equally inconsistent, and thus mostly unreliable. If influenza caused 630 deaths in the state, out of an estimated 500 000 who were sick (a relatively conservative estimate that one‐quarter were sick), the CFR would be 0.13%. In Hendricks County, which presented very specific numbers, 26 deaths from 3461 cases result in a CFR of 0.75%. In Adams County, which also presented specific numbers, 8 deaths from 300 cases result in a CFR of 2.67%, twenty times higher than estimated state rate. These estimates are consistent with claims that widespread illness contributed to increased death totals, even if these other studies have not attempted to calculate case fatality rates through a close study of mortality data, as is done here.^23^

**Figure 4:**
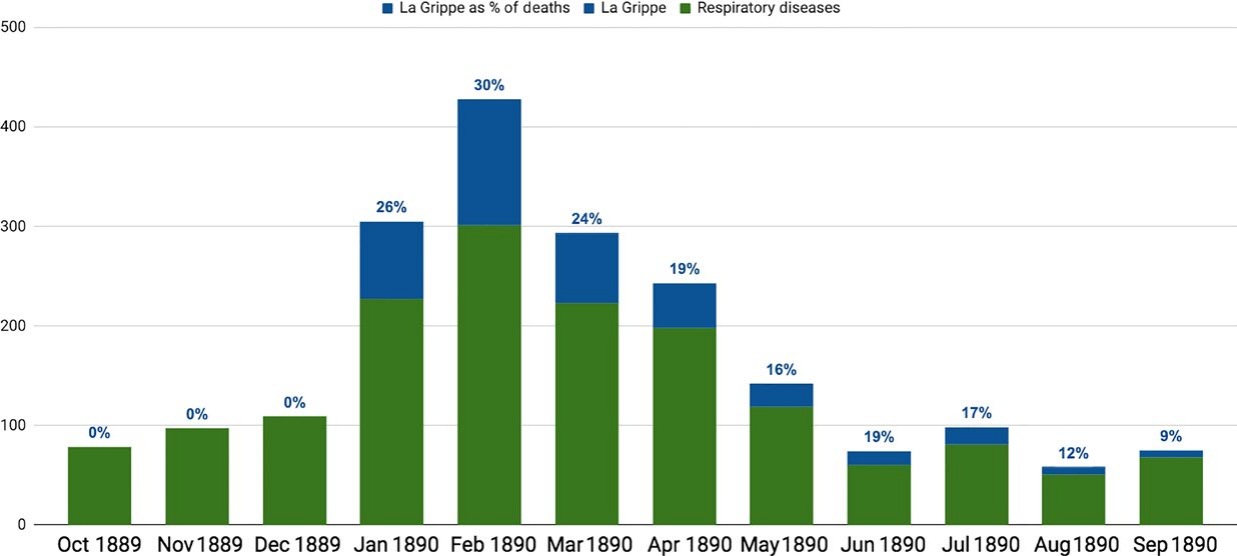
Indiana, Deaths from La Grippe and respiratory illnesses, 1890 Report^10^

As recognized by several health officers as well as the individual deaths recorded in newspapers cited above, deaths attributed specifically to la grippe represented just a fraction of deaths associated with the epidemic. The Board of Health statistics indicate that deaths from influenza in combination with other respiratory diseases spiked dramatically in the first 3 months of 1890 (Figure 4).^10^ The quantitative evidence confirms observations of county health officers that a dramatic change was being taking place, even if they were reluctant to specify exact numbers of cases or deaths.

**Figure 5:**
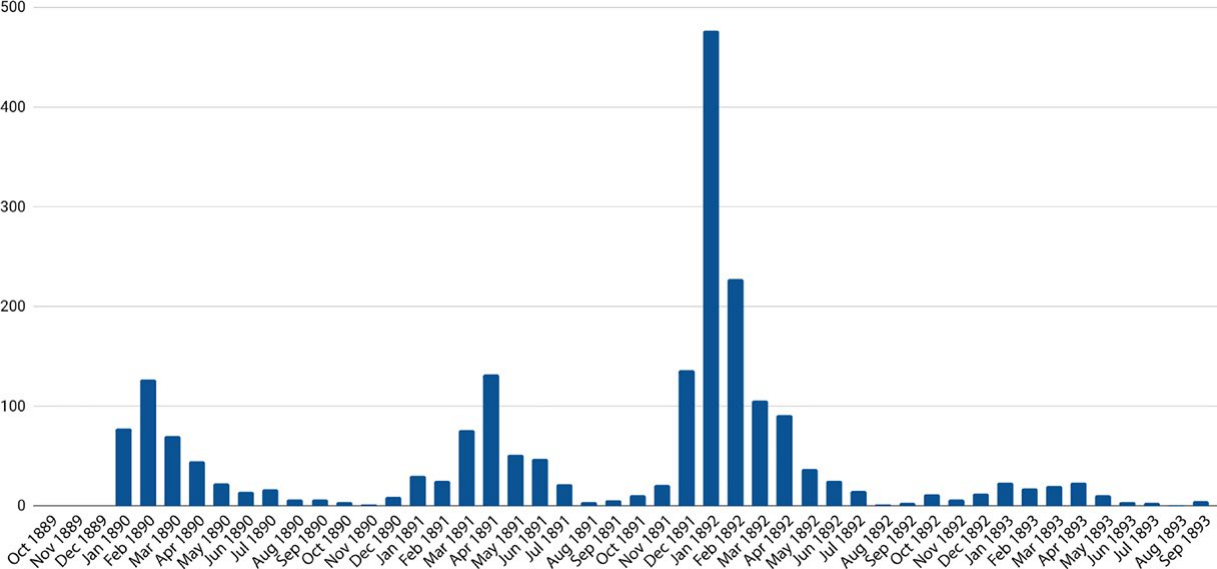
Indiana, La Grippe Deaths monthly, October 1889 to September 1893^10^

**Figure 6:**
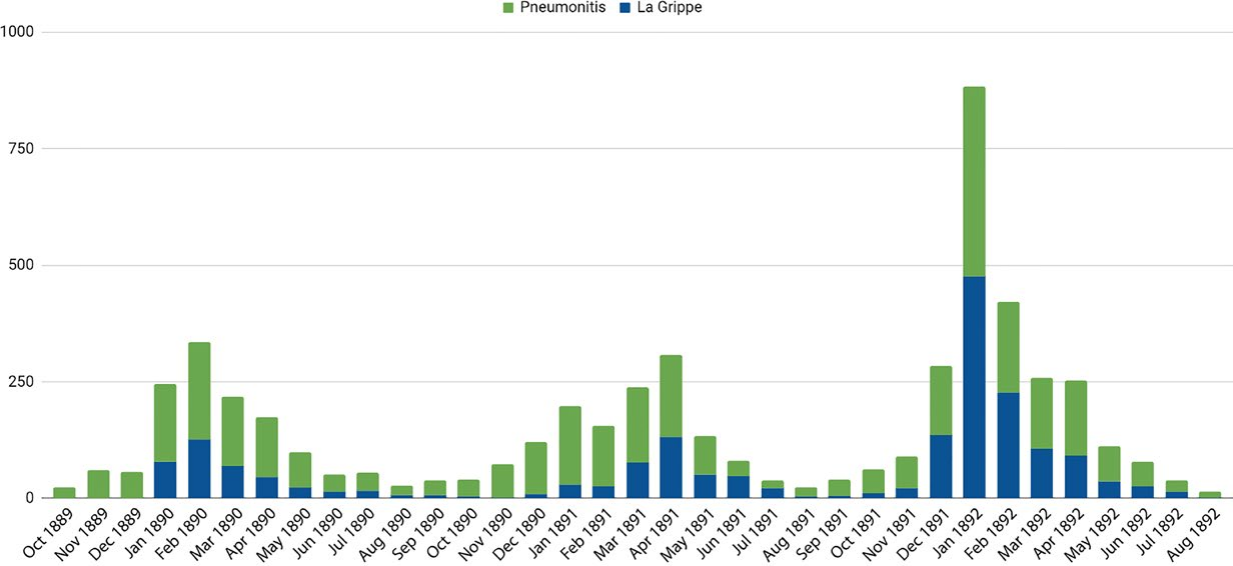
Deaths from La Grippe and Pneumonitis, October 1889 to September 1893^10^, ^33^, ^41^

Yet any historical interpretation of the 1890 influenza epidemic also requires considering developments in following years. In fact, the number of deaths actually increased following the outbreak: 409 in 1891 and 1152 in 1892.^10^, ^33^, ^41^ Figure 5, showing the monthly totals for these 3 years, illustrates how the deaths during the peak period of the 1890 epidemic (127 deaths recorded in February 1890) compared to subsequent months, five of which recorded more deaths: April 1891 (132), December 1891 (136), January 1892 (477), and February 1892 (228). The January 1892 total was almost seven times higher than the 77 deaths recorded in January 1890. Figure 6, combining deaths from pneumonitis and la grippe, confirms how much influenza‐associated deaths in early 1892 exceeded 1890 totals. These patterns were consistent with reports from other regions, which also indicated an increase in total deaths from influenza‐associated causes in 1891 and 1892.^24^, ^26^

The reference in the Fulton county health reports to deaths among “the aged and infirm” calls attention to the distribution of deaths by age category. As indicated in Table 1, deaths from influenza in the period beginning in 1890 were broadly consistent with the pattern evident in prior years (and still evident today) with highest death rates among children under 10 and the elderly.^26^ The death rates for adults aged 20‐50 years showed little change over these 5 years. The consistency of these patterns provides further evidence for the perception of influenza as relatively mild because the increased total deaths occurred mostly within established categories.

**Table 1:** Death rates by age‐group, respiratory diseases and influenza, 1888 to 1892 9, 10, 33, 41

Year	<10 y	10‐20 y	20‐30 y	30‐40 y	40‐50 y	50‐60 y	60‐70 y	>70 y
1888	1.35	0.36	0.51	0.65	1.01	1.30	2.98	6.77
1889	1.32	0.23	0.27	0.39	0.47	0.78	1.77	4.66
1890	1.26	0.26	0.47	0.57	0.68	1.12	2.10	5.88
1891	1.36	0.26	0.41	0.53	0.68	1.01	2.62	5.88
1892	1.39	0.28	0.59	0.76	1.11	2.07	5.01	14.48

Although the number of total deaths and particularly the number of influenza‐associated deaths spiked in spring 1891 and rose even higher in early 1892, county boards of health expressed little concern about the disease. Of the more than fifty county reports included in the 1891 report, just fourteen mentioned influenza, which is only half as many as reported on this disease a year earlier, when the disease was something unexpected.^33^ One year later, in the 1892 report, only one‐third of county health officers referred to influenza, even though the 811 deaths from this single cause were three times higher than the 1890 totals.^41^ It appears that county health officers, just like newspapers reporters and the general public, found a new disease outbreak more compelling than one that was expected, even if more deadly.

**Figure 7:**
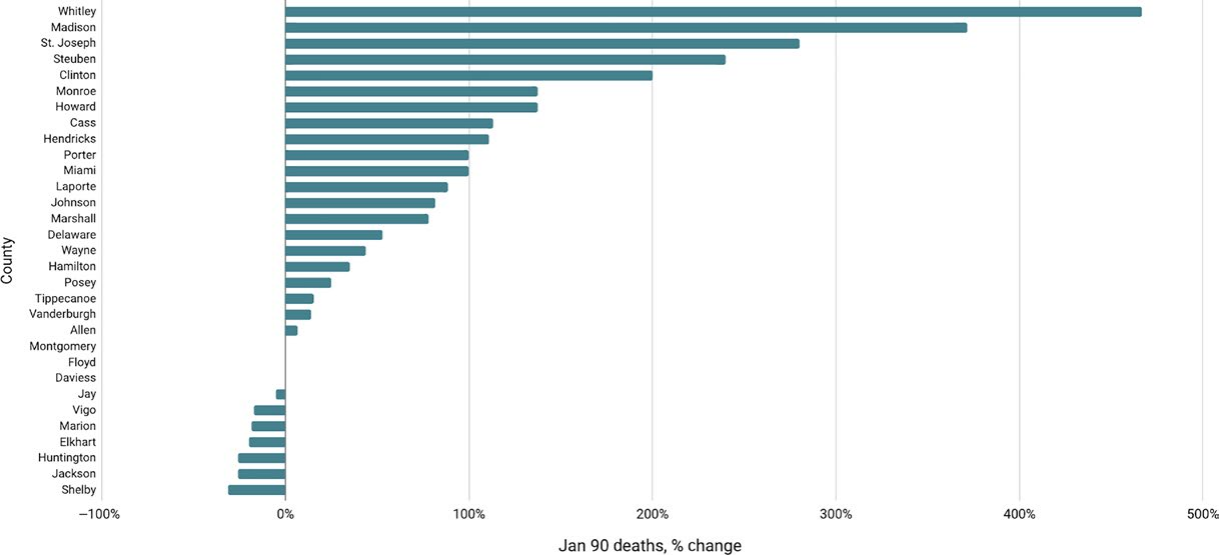
January 1890 Deaths as percentage of January 1889 Deaths, for largest counties^9^, ^10^

Given these patterns, it is easy to conclude that county health boards overreacted when they described the widespread effects of influenza in early 1890. Yet quantitative evidence also confirms that health officers had reason to be concerned. As shown in Figure 7, which compares the death toll in January 1890 to the same total in January 1889 for the largest counties in the state (to ensure meaningful comparisons), two‐thirds recorded more deaths than they had 1 year earlier—and in many counties, the total had doubled, tripled, or more.^9^, ^10^ But several large counties, including Marion County, the location for Indianapolis, actually recorded fewer deaths in January 1890 than in the same period a year earlier, which explains why overall deaths for Indiana showed little change, increasing just 1% from 1379 deaths in January 1889 to 1395 deaths in January 1890.

**Figure 8:**
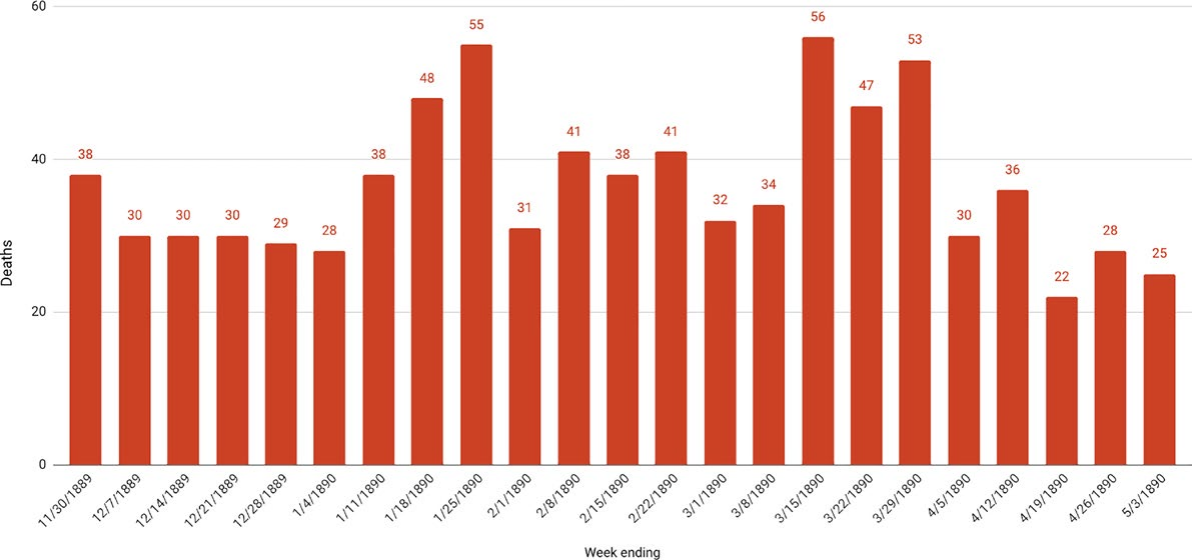
Deaths, Indianapolis, Weekly, November 1889 to May 1890^42^

Further evidence for the importance of understanding the context in which statistics were gathered, reported, and evaluated, particularly when it came to very high death rates, can be seen by a pattern of deaths from all causes in Indianapolis over a 6‐month period (Figure 8).^42^ The January 26, 1890 *Indianapolis Journal* article cited above was published at the moment death rates spiked—thus the accurate reference to a “sudden increase”—yet in the following week, deaths suddenly began to decrease, falling to a weekly total of 31. The 5 weeks that followed showed significant variation but not major increases. When death rates spiked again, in March 1890, perhaps indicating that some victims were suffering a second onset of illness, reaching a total actually exceeding the peak in January, it did not prompt any apparent concern, as the same newspaper referenced an increase in consumption deaths, but without further commentary.^43^ One of the significant contributions of historical epidemiology is placing both statistical evidence and interpretive statements in a broader context, thus recognizing the distinctiveness of critical moments while placing them in relationship to long‐term patterns. It was quite appropriate for the editors of the *Indianapolis Journal* and county health officers to comment on the unusual nature of the La Grippe epidemic, even as their own statistics can be used to indicate how these peak moments related to broader patterns.

Based on the above evidence and the critical analysis of reliability, consistency, and accuracy, it is reasonable to estimate that in Indiana, over 3 years beginning in January 1890, approximately 3200 people died of influenza and 11 700 people died of influenza and other respiratory diseases. These estimated totals are calculated using the number of deaths recorded by the State Board of Health from January 1890 to December 1892 for disease categories Respiratory (all) and La Grippe (listed under Miasmatic), with adjustments recognizing that state records included just over one‐half (62%) of the deaths reported by the census.^10^, ^32^, ^33^, ^41^

These estimated totals indicate a mortality rate per 1000 population from just influenza of 0.30 (1890), 0.41 (1891), and 0.75 (1892) and for influenza‐associated deaths of 1.56 (1890), 1.64 (1891), and 2.15 (1892). These rates are consistent with broad estimates for European countries such as France (1.6) and Germany (1.3) of deaths from “influenza and its complications” per 1000 population during and after the initial outbreak.^26^ Applying the Indiana rates to the US population of 62 million produces an estimated number of influenza victims of just under 100 000 and respiratory diseases and influenza victims of more than 300 000 during these 3 years. These estimates also suggest that the number of influenza victims increased in 1891 and 1892, but sporadically, without prompting the same alarm as in the first months of 1890, when “La Grippe or Russian influenza made its appearance and swept over the entire State.”

The death totals associated with influenza in the early 1890s never reached the levels that would appear during the Spanish Influenza, when influenza and pneumonia claimed more than 17 000 lives in Indiana in 1918 and 1919, accounting for more than one‐fifth of deaths from all causes.^29^, ^30^ The fact that mortality patterns by age categories remained within expected patterns in 1890 provides another important contrast to the 1918 Spanish Flu, when mortality rates rose unexpectedly among the age‐groups from 20 to 40.^27^ As this study has suggested, however, the significant increase in deaths from influenza that began in 1890 is essential for understanding both the later, more deadly, epidemic and current perspectives within epidemiology regarding influenza‐associated diseases, which remain among the ten most significant causes of death, despite a continued perception of influenza as not particularly threatening.^13^ The more experts, physicians, and the public understand about previous epidemics, this study argues, the more likely future outbreaks will be met with reasoned perception and factual knowledge.

This estimate of deaths nationally presumes that death rates among other populations are broadly similar to Indiana's death rates, as reported by the Board of Health and critically interpreted in this study. The best way to test this estimate, for the US and on a global scale, is to undertake a similarly detailed and critical examination of records from national health departments, regional, state, and municipal boards of health, newspapers, and medical journals, across a broad geographical span, in order to more accurately answer the important historical and epidemiological question of how many deaths resulted from the Russian influenza epidemic.
